# Association of Fibroblast Growth Factor 23 and Cardiac Mechanics in the Cardiovascular Health Study

**DOI:** 10.34067/KID.0000000643

**Published:** 2024-11-19

**Authors:** Keertana Jain, Ronit Katz, Tamara Isakova, Jorge R. Kizer, Shilpa Sharma, Bruce M. Psaty, Sanjiv J. Shah, Joachim Ix, Rupal Mehta

**Affiliations:** 1 Feinberg School of Medicine, Northwestern University, Chicago, Illinois; 2 Collaborative Health Studies Coordinating Center, Department of Biostatistics, University of Washington, Seattle, Washington; 3 Division of Nephrology, Department of Medicine, Feinberg School of Medicine, Northwestern University, Chicago, Illinois; 4 Cardiology Section, San Francisco Veterans Affairs Health Care System, Departments of Medicine, Epidemiology and Biostatistics, University of California San Francisco, San Francisco, California; 5 Department of Medicine, David Geffen School of Medicine at UCLA, Los Angeles, California; 6 Departments of Medicine, Epidemiology, and Health Systems and Population Health, University of Washington, Seattle, Washington; 7 Division of Cardiology, Department of Medicine, Northwestern University, Feinberg School of Medicine, Chicago, Illinois; 8 Nephrology Section, Veterans Affairs San Diego Healthcare System, La Jolla, California; 9 Division of Nephrology, Department of Medicine, Jesse Brown Veterans Affairs Health Administration, Chicago, Illinois

**Keywords:** cardiovascular disease, echocardiography, kidney disease, mineral metabolism, risk factors

## Abstract

**Key Points:**

- Evaluation of cardiac mechanics through two-dimensional speckle tracking echocardiography can identify early alterations in cardiac function.
- In a subset of the Cardiovascular Health Study, c-terminal and intact fibroblast growth factor 23 were not independently associated with cardiac mechanic indices.

**Background:**

Elevated levels of fibroblast growth factor 23 (FGF23) are associated with left ventricular hypertrophy and heart failure in individuals with and without kidney disease. Prior studies investigated the association of FGF23 and structural cardiac changes using conventional echocardiography, which is limited in its ability to detect early cardiac dysfunction. We investigated the relationship between FGF23 levels and cardiac dynamics using two-dimensional speckle tracking echocardiography (2D-STE), a novel imaging modality.

**Methods:**

This was a cross-sectional analysis of data from the Cardiovascular Health Study (CHS), an ongoing prospective, population-based cohort study. The study population included 506 participants from CHS with available c-terminal FGF23 (cFGF23) and intact FGF23 (iFGF23) measurements from 1996 to 1997 and 2D-STE images from 1994 to 1995. Forty-two percent of the study population had CKD, defined as an eGFR <60 ml/min per 1.73 m^2^, and the mean eGFR was 63 ml/min per 1.73 m^2^. The primary exposures were cFGF23 and iFGF23. The primary outcomes were six 2D-STE parameters performed at the 1994–1995 study visit. Linear regression models were used to examine the independent associations of FGF23 with six cardiac 2D-STE indices adjusting for demographics, cardiovascular risk factors, markers of kidney disease severity, and inflammation.

**Results:**

cFGF23 levels were moderately correlated with iFGF23 levels in the CHS population. In fully adjusted models, cFGF23 was associated with left atrial dysfunction, but no other cardiac imaging parameter (*β* estimate, −2.47; 95% confidence interval, −4.68 to −0.25; Table 2). iFGF23 was not associated with any of the six 2D-STE indices. Limitations include small sample size and noncurrent FGF23 measurements and 2D-STE imaging.

**Conclusions:**

In a limited sample of individuals enrolled in the CHS with cFGF23 and iFGF23 measurements, we did not find consistent associations between FGF23 levels and 2D-STE parameters. Further investigations in a larger population with concurrent 2D-STE are needed to better understand the associations of FGF23 with early changes in cardiac mechanics.

## Visual Abstract

**Figure:**
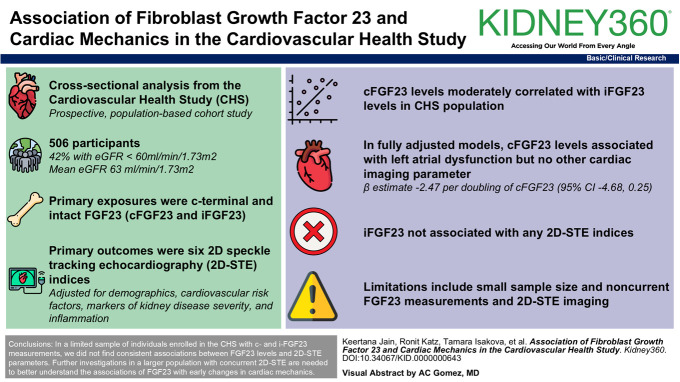

## Introduction

Individuals with CKD experience increased rates of cardiovascular disease (CVD) and heart failure (HF) compared with the general population.^1^ As CKD progresses, the risk of death and hospitalizations from HF rises.^2^ Numerous prior investigations have focused on understanding and inhibiting potential pathways that can lead to increased risk of HF in patients with CKD. Proposed mechanisms include shared risk factors such as hypertension (HTN) and diabetes.^1,3,4^ Inflammation, volume overload, anemia, oxidative stress, and upregulation of the renin-angiotensin-aldosterone system and sympathetic nervous system also contribute to HF pathogenesis.^1,3,4^ These factors explain some, but not all, of the heightened HF risk in patients with CKD.

Alterations in parameters of mineral metabolism are ubiquitous in CKD and are also strongly associated with HF.^5^ Fibroblast growth factor-23 (FGF23) is an osteocyte-derived hormone that regulates phosphate and vitamin D homeostasis. In CKD populations, higher FGF23 is associated with risk of HF independent of kidney function and traditional CVD risk factors.^5^ A direct pathologic role for FGF23 in promoting HF pathogenesis is supported by preclinical studies that demonstrate FGF23 binds to fibroblast growth factor receptor 4 on cardiac myocytes and induces left ventricular hypertrophy.^6^ Blocking fibroblast growth factor receptor 4 can ameliorate left ventricular hypertrophy in preclinical models.^6^ FGF23 may also contribute to myocardial fibrosis and inflammation, leading to impaired leukocyte recruitment, which, in turn, may promote the development of HF.^6–10^

Most prior studies in humans that investigated the association of FGF23 with structural cardiac abnormalities used conventional two-dimensional echocardiograms. Conventional echocardiography is limited in its ability to detect abnormalities early in the progression of myocardial dysfunction. Two-dimensional speckle tracking echocardiography (2D-STE) provides a more sensitive tool than conventional echocardiography by directly tracking myocardial motion and deformation processes and provides detailed assessments of regional and global systolic and diastolic function.^11^ It allows for a complete characterization of both left and right ventricular myocardial contractility beyond depiction of myocardial geometric changes in size and volume.^12^ For example, left ventricular longitudinal strain (LVLS) measures the maximal shortening of the left ventricular longitudinal myocardial segments during systole.^13^ Left atrial reservoir strain (LARS) can assess the ability of the left atrium to fill during systole.^13^ Reduced absolute LVLS demonstrates abnormal left ventricular (LV) systolic function, and reduced absolute LARS suggests reduced compliance of the left atrium.^13^ Importantly, prior data demonstrate that abnormalities appreciated on 2D-STE imaging, such as reduced absolute LVLS and LARS, are important independent risk factors of HF, even in the absence of reduced ejection fraction or left ventricular hypertrophy.^14–17^

A prior study in the Cardiovascular Health Study (CHS) showed associations between c-terminal FGF23 (cFGF23) and greater left ventricular mass and left ventricular hypertrophy, with stronger relationships in participants with CKD.^18^ In this study, we investigated the associations of plasma FGF23 with multiple indices of cardiac mechanics identified through 2D-STE in participants enrolled in the CHS. We hypothesized that higher intact FGF23 (iFGF23), which represents the biologically active hormone, and cFGF23, which represents both the biologically active hormone and post-translational c-terminal fragments, were independently associated with worse indices of cardiac mechanics on 2D-STE.

## Methods

### Study Population

CHS is an ongoing, prospective, population-based cohort study conducted to investigate risk factors of CVD in adults aged 65 years and older.^19^ The study recruited both White and Black adults during two time periods spanning from 1989 to 1993 across four communities in the United States (Forsyth County, NC; Sacramento County, CA; Washington County, MD; and Pittsburgh, PA).^19^ The original cohort recruited 5201 individuals during 1989–1990, with an additional 687 individuals recruited during 1992–1993 as part of a supplemental Black cohort.^19^ Participants had annual in-person study visits and were contacted by phone every 6 months. Each respective Institutional Review Board approved the protocols, and all participants gave written informed consent.

Plasma cFGF23 was measured among 3107 of the 3406 participants who participated in the year 9 (1996–1997) examination.^20^ Among these, we subsequently randomly selected a subcohort of 1000 participants for additional mineral metabolism measurements (serum calcium, phosphate, intact parathyroid hormone, 25-hydroxyvitamin D, and iFGF23), among whom 844 participants had complete data for this analysis.

For our cross-sectional analyses studying FGF23 with 2D-STE parameters, we excluded individuals with a history of prevalent HF at the year 9 (1996–1997) study visit (*N*=76). Individuals were also excluded if they were missing 2D-STE data from the year 7 (1994–1995) study visit (*N*=262). The total cardiac subcohort population for our cross-sectional study therefore comprised 506 participants.

### Primary Exposures

The primary exposures were cFGF23 and iFGF23. Specimens for plasma cFGF23 were collected after an 8-hour fast at the year 9 (1996–1997) study visit.^20^ Specimens were stored at −70°C until 2010 when they were thawed. cFGF23 was measured using a second-generation C-terminal assay (Immutopics, San Clemente, CA) at the University of Vermont. The intra-assay and interassay coefficients of variation (CVs) for cFGF23 ranged from 7.4% to 10.6%. Plasma iFGF23 concentrations were measured in previously unthawed EDTA plasma using Kainos ELISA (Kainos Laboratories). The intra-assay and interassay CVs ranged from 5.0% to 8.7%.^20,21^

### Outcomes

The primary outcomes were six 2D-STE parameters performed at the year 7 (1994–1995) study visit. Parameters included LVLS, left ventricular early diastolic strain rate (EDSR), left ventricular early diastolic tissue velocity (e′ velocity), E/e′ ratio, right ventricular free wall strain (RVFWS), and LARS. Lower absolute LVLS (represents left ventricular systolic function), EDSR (represents left ventricular diastolic function), RVFWS (represents right ventricular systolic function), and LARS (represents left atrial function) denote more abnormal deformation strain patterns. A lower e′ velocity represents more impaired left ventricular relaxation during diastolic relaxation.^22^ A higher E/e′ ratio represents increased left ventricular filling pressures, also a measure of diastolic dysfunction.^22^ Importantly, 2D-STE often underestimates e′ velocities compared to standard tissue Doppler imaging measurements. This is reflected by lower e′ and higher E/e′ ratios in studies using 2D-STE measurements when compared with prior studies using standard tissue Doppler imaging measurements.^23^

Comprehensive two-dimensional, M-mode, and Doppler echocardiograms were obtained at the year 7 (1994–1995) study visit.^24^ Technicians recorded echocardiograms onto Super video home system tapes using Toshiba SSH-160A cardiac ultrasound machines. Video home system echocardiogram images were digitized at the Echocardiography Reading Center (Irvine, CA, for the original cohort, and Washington, DC, for the African American cohort) between 2016 and 2018.^24^ Archived CHS echocardiograms were transformed into digital format using the TIMS 2000 DICOM system (Foresight Imaging, Chelmsford, MA), as previously described.^23^ Cine loops of 2–4 cardiac cycles were digitized at a frame rate of 30 frames per second (fps) from the parasternal short-axis and apical two-, three-, and four-chamber view. The apical four-chamber view is representative of all three apical views combined and was used for this report.

Five experienced readers performed speckle tracking echocardiography on the digitized images to obtain strain parameters using TOMTEC Cardiac Performance Analysis, v4.5 software. A blinded reader assigned each echocardiogram an image quality score, as described previously.^23^ Readers manually traced the left and right ventricles and left atrial endocardial borders in the apical four-chamber view to obtain LVLS, EDSR, e′ velocity, RVFWS, and LARS curves. e′ Velocity was derived as the average of the septal and lateral mitral annulus tissue velocities. Reproducibility of CHS strain measures have previously been reported with good reproducibility and low bias.^23–28^ We present all 2D-STE strain rates as absolute positive percentages.

### Covariates

Information on patient demographics, medical history, and clinical data were collected at the year 9 (1996–1997) study visit, concurrent with FGF23 measurement. Self-reported covariates included sex, race, education, physical activity, current smoking, and alcohol use. Presence of diabetes was defined by self-report, use of hypoglycemic medications or insulin, or a fasting blood glucose ≥126 mg/dl.^29^ Therapy for HTN was ascertained through medication use because participants were asked to bring their medications to clinic visits. CVD was defined as adjudicated coronary heart disease (angina pectoris, myocardial infarction, angioplasty, or coronary bypass surgery) or stroke before the year 9 (1996–1997) study visit.^30^ Cystatin C values were measured using a BNII nephelometer, and eGFR was calculated using the CKD Epidemiology Collaboration equation.^31^ Urine albumin and urine creatinine were measured from morning samples using a Kodak Ektachem 700 Analyzer to calculate urine albumin–creatinine ratio (UACR). Given the relationship between iron stores and CVD and that iron deficiency itself is a stimulus for FGF23 transcription, measurements of iron homeostasis were included as covariates.^32^ Ferritin was measured in EDTA plasma using a Beckman Coulter clinical analyzer. 2D-STE reader, CHS site, and quality were also included as covariates.

### Statistical Analyses

We first compared year 9 (1996–1997) study visit characteristics of the 506 individuals included in this report with the original and minority CHS study population who had available measurements of eGFR and UACR (*N*=3292). We then used standard descriptive statistics to compare demographics and clinical characteristics of our study population (*n*=506) according to cFGF23 and iFGF23 quartiles.

We used Spearman correlation to evaluate the strength and direction of the relationship between cFGF23 and iFGF23, both with each other and with each of the six cardiac 2D-STE parameters. We next used multivariable linear regression models to investigate the independent associations of cFGF23 and iFGF23 with each of the six cardiac 2D-STE parameters. FGF23 levels were expressed both as a continuous variable per doubling of value and in quartiles. We hierarchically adjusted for main effects of imaging (2D-STE reader, quality, and CHS site), demographics and CVD risk factors (age, sex, race, education, physical activity, smoking, body mass index, use of HTN medications, systolic BP, prevalent CVD, and alcohol use), markers of kidney severity and mineral metabolism markers (eGFR, UACR, calcium, phosphorus, parathyroid hormone, and 25-hydroxyvitamin D), and measurements of inflammation and iron stores (C-reactive protein [CRP], ferritin, and transferrin saturation). Given the findings, we present condensed models, with model 1 adjusted for main effects of imaging and model 2 adjusted for all other covariates. Multiplicity testing was not performed, and our results should be regarded as exploratory. Analyses were performed using SAS version 9.4 (Cary, NC). Two-sided *P* values < 0.05 were considered statistically significant.

## Results

Baseline characteristics from year 9 (1996–1997) of the cardiac subcohort included for this report (*N*=506) compared with the total CHS cohort with kidney function measurements (*N*=3292) are presented in Supplemental Table 1. The mean age was 78 years in both groups, and the mean eGFR was 63. The percentage of female participants was similar in both groups. The study population for this report had a lower percentage of Black participants (10% versus 15%) and participants with diabetes (12% versus 14%). Median iFGF23 and cFGF23 concentrations were 61 (interquartile range [IQR], 48–79) pg/ml and 68 (IQR, 53–92) relative units/ml for the cardiac subcohort and 61 (IQR, 48–78) pg/ml and 70 (IQR, 53–99) relative units/ml for the total CHS cohort, respectively.

Table 1 presents baseline characteristics of the total population in this report, categorized according to cFGF23 quartiles. The mean age was 78 (SD, 4) years, with 61% of individuals being female, and the mean eGFR was 63 ml/min per 1.73 m^2^ (SD, 18). Forty-two percent of individuals had CKD defined by an eGFR of <60 ml/min per 1.73 m^2^. The percentage of individuals with an eGFR of <60 ml/min per 1.73 m^2^ increased linearly from quartile 1 to quartile 4. In addition, a higher prevalence of diabetes was observed in quartile 4 compared with that in quartile 1. Furthermore, individuals in quartile 4 had higher incidence of UACR ≥30 mg/g compared with those in quartile 1. Ferritin levels demonstrated a linear decrease from quartile 1 to quartile 4, whereas CRP levels increased from quartile 1 to quartile 4.

**Table 1:** Demographics and clinical characteristics by quartiles of cFGF23 in the cardiac subcohort

Baseline Characteristics	Subcohort (*n*=506)	Q1 ≤53 RU/ml *n*=132	Q2 53–69 RU/ml *n*=128	Q3 70–95 RU/ml *n*=133	Q4 >95 RU/ml *n*=113
Age, yr (SD)	78 (4)	79 (4)	77 (5)	78 (4)	79 (5)
Female sex, No. (%)	310 (61)	69 (52)	73 (57)	91 (68)	77 (68)
Black, No. (%)	51 (10)	16 (12)	18 (14)	7 (5)	10 (9)
**Education, No. (%)**					
< High school	73 (15)	13 (10)	17 (13)	23 (17)	20 (18)
High school graduate	209 (41)	49 (37)	58 (45)	59 (44)	43 (38)
Post-secondary	223 (44)	69 (53)	53 (41)	51 (38)	50 (44)
**Smoking status, No. (%)**					
Never	238 (48)	67 (52)	63 (50)	57 (44)	51 (64)
Former	220 (44)	57 (44)	54 (43)	61 (47)	48 (44)
Current	38 (8)	5 (4)	10 (8)	12 (9)	11 (10)
Alcohol use (IQR)	0 (0,1)	0 (0,1)	0 (0,1)	0 (0,1)	0 (0,1)
BMI, kg/m^2^ (SD)	26.8 (4.4)	26.3 (4.3)	26.7 (4.1)	26.6 (4.0)	27.8 (5.1)
DM, No. (%)	61 (12)	9 (7)	16 (13)	22 (17)	14 (12)
SBP, mm Hg (SD)	137 (22)	137 (22)	138 (22)	136 (21)	138 (22)
HTN meds, No. (%)	266 (53)	48 (36)	67 (52)	77 (58)	74 (66)
CRP, mg/L (IQR)	2.22 (1.02–4.89)	1.82 (0.91–4.20)	1.79 (0.91–4.34)	2.32 (1.14–5.06)	2.96 (1.56–6.34)
eGFR, ml/min per 1.73 m^2^, (SD)	63 (18)	73 (15)	67 (17)	62 (15)	49 (18)
eGFR <60 ml/min per 1.73 m^2^, No. (%)	214 (42)	26 (20)	46 (36)	57 (43)	85 (75)
UACR, mg/g (IQR)	8 (5–19)	7 (5–12)	8 (5–17)	8 (5–20)	10 (5–34)
25-hydroxyvitamin D, ng/ml	29 (11)	30 (10)	28 (12)	29 (11)	29 (12)
Ferritin, ng/ml (IQR)	103 (57–176)	125 (75–202)	117 (65–187)	94 (54–174)	67 (30–121)
Transferrin saturation (SD)	31 (10)	33 (10)	32 (9)	30 (10)	26 (10)
Calcium, mg/dl (SD)	9.8 (0.6)	9.7 (0.5)	9.8 (0.6)	9.9 (0.6)	9.8 (0.6)
Phosphorus, mg/dl (SD)	3.8 (0.6)	3.7 (0.6)	3.8 (0.6)	3.9 (0.6)	4.0 (0.5)
PTH, pg/ml (IQR)	41 (33–55)	38 (31–50)	43 (30–55)	41 (33–52)	48 (36–65)

BMI, body mass index; cFGF23, c-terminal fibroblast growth factor 23; CRP, C-reactive protein; DM, diabetes mellitus; HTN, hypertension; IQR, interquartile range; PTH, parathyroid hormone; RU, relative units; SBP, systolic BP; UACR, urine albumin–creatinine ratio.

Supplemental Table 2 presents the baseline characteristics of the total population in this report categorized by iFGF23 quartiles. The percentage of individuals with an eGFR of <60 ml/min per 1.73 m^2^, and median albuminuria exhibited a linear increase from quartile 1 to quartile 4. Ferritin and CRP levels were higher in quartile 4 compared with quartile 1. In addition, a higher proportion of individuals in quartile 4 had a UACR ≥30 compared with those in quartile 1.

### Correlation Analyses

**Figure 1:**
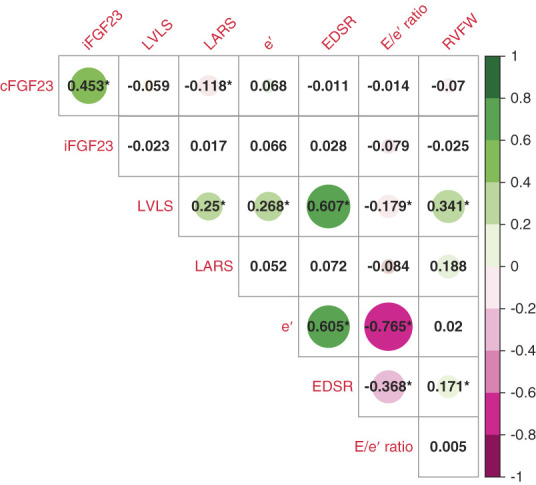
**Spearman coefficient correlation matrix demonstrating correlation of cFGF23 and iFGF23 with cardiac 2D-STE parameters.** *Correlation is significant at the 0.01 level (two-tailed). cFGF23, c-terminal fibroblast growth factor 23; e′, LV early diastolic tissue velocity; EDSR, early diastolic strain rate; iFGF23, intact fibroblast growth factor 23; LARS, left atrial reservoir strain; LV, left ventricular; LVLS, left ventricular longitudinal strain; RVFWS, right ventricular free wall strain; 2D-STE, two-dimensional speckle tracking echocardiography.

CFGF23 was moderately correlated with iFGF23 (Spearman coefficient, 0.453; *P* < 0.01). Figure 1 presents correlations of cFGF23 and iFGF23 with the six cardiac 2D-STE parameters. cFGF23 was significantly correlated with LARS (Spearman coefficient, −0.118; *P* < 0.01), but not with any other cardiac parameter. iFGF23 was not significantly correlated with any of the 2D-STE parameters.

### Linear Regression Analyses

When expressed as a continuous variable, cFGF23 showed a significant association with LARS in the fully adjusted linear regression model (per doubling of cFGF23, *β* estimate, −2.47; 95% confidence interval [CI], −4.68 to −0.25; Table 2). In quartile analysis, cFGF23 quartile 4 was significantly associated with LARS after adjustment for demographics, CVD, and kidney risk factors (*β* estimate, −5.99; 95% CI, −10.80 to −1.18; Table 2). cFGF23 was not independently associated with any other cardiac mechanics parameter.

**Table 2:** Association of cFGF23 cardiac mechanics measured by 2D-STE

2D-STE Parameters	Model 1	Model 2
	*β* (95% CI)	*β* (95% CI)
**LVLS**		
cFGF23 (doubling)	−0.25 (−0.60 to 0.10)	−0.25 (−0.70 to 0.21)
Quartiles		
*Q1 (<53)*	Ref	Ref
*Q2 (53–59)*	0.47 (−0.28 to 1.22)	0.48 (−0.30 to 1.26)
*Q3 (70–95)*	−0.21 (−0.96 to 0.53)	−0.17 (−0.97 to 0.64)
*Q4 (>95)*	−0.39 (−1.17 to 0.40)	−0.33 (−1.31 to 0.65)
**LARS**		
cFGF23 (doubling)	−1.35 (−3.08 to 0.38)	**−2.47 (−4.68 to −0.25)^a^**
Quartiles		
*Q1 (<53)*	Ref	Ref
*Q2 (53–59)*	−0.19 (−3.85 to 3.48)	−0.59 (−4.38 to 3.20)
*Q3 (70–95)*	0.14 (−3.53 to 3.80)	−0.92 (−4.86 to 3.01)
*Q4 (>95)*	−3.42 (−7.31 to 0.46)	**−5.99 (−10.80 to −1.18)^a^**
**e′**		
cFGF23 (doubling)	**0.11 (0.01 to 0.21)^a^**	0.05 (−0.08 to 0.18)
Quartiles		
*Q1 (<53)*	Ref	Ref
*Q2 (53–59)*	0.05 (−0.16 to 0.26)	0.02 (−0.20 to 0.24)
*Q3 (70–95)*	0.17 (−0.03 to 0.41)	0.11 (−0.11 to 0.34)
*Q4 (>95)*	0.19 (−0.03 to 0.41)	0.06 (−0.21 to 0.34)
**EDSR**		
cFGF23 (doubling)	0.01 (−0.01 to 0.04)	0.01 (−0.02 to 0.04)
Quartiles		
*Q1 (<53)*	Ref	Ref
*Q2 (53–59)*	0.01 (−0.04 to 0.06)	0.02 (−0.04 to 0.06)
*Q3 (70–95)*	0 (−0.05 to 0.05)	0 (−0.05 to 0.05)
*Q4 (>95)*	0.01 (−0.04 to 0.06)	0.01 (−0.05 to 0.07)
**E/e′**		
cFGF23 (doubling)	−0.85 (−2.58 to 0.89)	−0.10 (−2.37 to 2.18)
Quartiles		
*Q1 (<53)*	Ref	Ref
*Q2 (53–59)*	−0.50 (−4.26 to 3.26)	0.11 (−3.83 to 4.05)
*Q3 (70–95)*	−1.48 (−5.24 to 2.29)	−0.58 (−4.64 to 3.48)
*Q4 (>95)*	−1.24 (−5.18 to 2.69)	0.74 (−4.20 to 5.67)
**RVFWs**		
cFGF23 (doubling)	−0.13 (−0.65 to 0.40)	0.26 (−0.42 to 0.94)
Quartiles		
*Q1 (<53)*	Ref	Ref
*Q2 (53–59)*	−0.09 (−1.22 to 1.04)	0.08 (−1.10 to 1.25)
*Q3 (70–95)*	−0.05 (−1.18 to 1.08)	0.25 (−0.98 to 1.48)
*Q4 (>95)*	−0.50 (−1.68 to 0.69)	0.04 (−1.45 to 1.53)

Model 1 adjusted for reader, quality, site, age, gender, race, education and physical activity. Model 2 adjusts for Model 1 and smoking, BMI, HTN medications, SBP, prevalent CVD, alcohol use, eGFR, UACR, Ca, Phosphorus, PTH, 25-hydroxyvitamin D, Ferritin, Transferrin Saturation and CRP. BMI, body mass index; Ca, calcium; cFGF23, c-terminal fibroblast growth factor 23; CI, confidence interval; CRP, C-reactive protein; CVD, cardiovascular disease; e', left ventricular early diastolic tissue velocity; EDSR, left ventricular early diastolic strain rate; HTN, hypertension; LARS, left atrial reservoir strain; LV, left ventricular; LVLS, left ventricular longitudinal strain; PTH, parathyroid hormone; RVFW, right ventricular free wall strain; SBP, systolic BP; 2D-STE, two-dimensional speckle tracking echocardiography; UACR, urinary albumin-creatinine ratio.

a Bold values denotes statistically significant.

When expressed as a continuous variable, iFGF23 was not significantly associated with any 2D-STE parameter in multivariable linear regression models (Table 3). In quartile analyses, quartile 3 was significantly associated with E/e′ in minimally adjusted models (*β* estimate, −4.61; 95% CI, −8.48 to −0.74; Table 3). However, after multivariable adjustment, this association was attenuated and no longer remained significant (*β* estimate, −3.33; 95% CI, −7.40 to 0.74; Table 3).

**Table 3:** Association of iFGF23 cardiac mechanics measured by 2D-STE

2D-STE Parameters	Model 1	Model 2
	*β* (95% CI)	*β* (95% CI)
**LVLS**		
iFGF23 (doubling)	0 (−0.49 to 0.49)	0.15 (−0.43 to 0.72)
Quartiles		
*Q1 (<48)*	Ref	Ref
*Q2 (48–61)*	0.08 (−0.67 to 0.84)	0.18 (−0.61 to 0.96)
*Q3 (62–78)*	0.10 (−0.68 to 0.88)	0.03 (−0.80 to 0.85)
*Q4 (>78)*	0.11 (−0.67 to 0.89)	0.29 (−0.59 to 1.17)
**LARS**		
iFGF23 (doubling)	0.17 (−2.2.4 to 2.58)	−0.97 (−3.79 to 1.85)
Quartiles		
*Q1 (<48)*	Ref	Ref
*Q2 (48–61)*	0.66 (−3.05 to 4.38)	0.15 (−3.70 to 4.00)
*Q3 (62–78)*	1.20 (−2.67 to 5.06)	0.01 (−4.06 to 4.09)
*Q4 (>78)*	1.69 (−2.16 to 5.54)	0.43 (−3.90 to 4.76)
**e′**		
iFGF23 (doubling)	0.10 (−0.03 to 0.24)	0.05 (−0.11 to 0.20)
Quartiles		
*Q1 (<48)*	Ref	Ref
*Q2 (48–61)*	0.04 (−0.17 to 0.25)	0.05 (−0.17 to 0.27)
*Q3 (62–78)*	0.23 (0.02 to 0.45)	0.17 (−0.06 to 0.40)
*Q4 (>78)*	0.14 (−0.07 to 0.36)	0.07 (−0.18 to 0.31)
**EDSR**		
iFGF23 (doubling)	0.02 (−0.01 to 0.05)	0.03 (−0.01 to 0.07)
Quartiles		
*Q1 (<48)*	Ref	Ref
*Q2 (48–61)*	0.03 (−0.02 to 0.07)	0.04 (−0.01 to 0.09)
*Q3 (62–78)*	0.05 (−0.003 to 0.10)	0.04 (−0.02 to 0.09)
*Q4 (>78)*	0.04 (−0.01 to 0.09)	0.05 (−0.004 to 0.11)
**E/e′**		
iFGF23 (doubling)	−0.35 (−2.74 to 2.05)	0.72 (−2.09 to 3.53)
Quartiles		
*Q1 (<48)*	Ref	Ref
*Q2 (48–61)*	−2.47 (−6.23 to 1.28)	−2.65 (−6.52 to 1.23)
*Q3 (62–78)*	**−4.61 (−8.48 to −0.74)^a^**	−3.33 (−7.40 to 0.74)
*Q4 (>78)*	−1.26 (−5.10 to 2.58)	−0.09 (−4.42 to 4.24)
**RVFWs**		
iFGF23	−0.05 (−0.78 to 0.69)	0.19 (−0.69 to 1.07)
Quartiles		
*Q1 (<48)*	Ref	Ref
*Q2 (48–61)*	−0.29 (−1.43 to 0.85)	−0.23 (−1.41 to 0.95)
*Q3 (62–78)*	−0.12 (−1.28 to 1.04)	−0.09 (−1.31 to 1.14)
*Q4 (>78)*	0.03 (−1.13 to 1.19)	0.34 (−0.99 to 1.67)

Model 1 adjusted for reader, quality, site, age, gender, race, education and physical activity. Model 2 adjusts for Model 1 and smoking, BMI, HTN medications, SBP, prevalent CVD, alcohol use, eGFR, UACR, Ca, Phosphorus, PTH, 25-hydroxyvitamin D, Ferritin, Transferrin Saturation and CRP. BMI, body mass index; Ca, calcium; CI, confidence interval; CRP, C-reactive protein; CVD, cardiovascular disease; e', left ventricular early diastolic tissue velocity; EDSR, left ventricular early diastolic strain rate; HTN, hypertension; iFGF23, intact fibroblast growth factor 23; LARS, left atrial reservoir strain; LV, left ventricular; LVLS, left ventricular longitudinal strain; PTH, parathyroid hormone; RVFW, right ventricular free wall strain; SBP, systolic BP; 2D-STE, two-dimensional speckle tracking echocardiography; UACR, urinary albumin-creatinine ratio.

a Bold values denotes statistically significant.

## Discussion

In a cohort of community-living older individuals with and without CKD enrolled in the CHS, we comprehensively investigated the association of cFGF23 and iFGF23 concentrations with cardiac mechanics indices on 2D-STE. The cardiac mechanics indices represented ventricular, atrial, and diastolic function. We demonstrate that cFGF23 was associated only with left atrial dysfunction, but no other index of cardiac mechanics. iFGF23 was not associated with any 2D-STE parameter. Overall, we did not find consistent associations between FGF23 concentrations and cardiac mechanics indices on 2D-STE in patients with and without CKD in the CHS.

Contrary to our results, prior studies often demonstrated significant associations between higher cFGF23 levels and CVD outcomes in individuals with and without CKD. Increased levels of cFGF23 were consistently associated with increases in LV mass and LV hypertrophy, including prior studies within the CHS.^18,33^ Elevated cFGF23 levels were also associated with mitral valve calcification.^34^ The independent associations of cFGF23 with CVD extend beyond structural abnormalities and also include clinical events. In the CHS, elevated levels of cFGF23 were associated with all-cause mortality and incident HF in patients with and without CKD.^20^ The risk relationships were notably stronger in the subgroup with CKD compared with individuals without CKD.^20^

Several studies have investigated the association of iFGF23 concentrations with CVD. iFGF23 levels were associated with cardiac hypertrophy and systolic dysfunction in patients with and without CKD.^35^ Elevated iFGF23 levels were associated with LV mass and an increased risk of LV hypertrophy in an elderly population, with stronger associations in CKD subgroups.^36^ In addition, elevated iFGF23 levels were also associated with cardiac valve calcification.^37^ In the Multi-Ethnic Study of Atherosclerosis (MESA), iFGF23 levels were associated with greater LV mass, coronary artery calcification, and increased rates of HF and coronary heart disease.^38^ In the Atherosclerosis Risk in Communities study, levels of iFGF23 above 40 pg/ml were associated with risk of incident coronary heart disease, HF, and CV mortality, independent of kidney function.^5^ Prior studies in the CHS demonstrated associations of both cFGF23 and iFGF23 with HF and death. These associations were independent of inflammation and iron deficiency, but adjustment for kidney function influenced the associations differently, such that the association of cFGF23 with these end points remained, whereas that of iFGF23 was diminished or no longer present.^21^

Few studies have explored the associations of FGF23 with early changes in systolic and diastolic dysfunction as measured through myocardial cardiac strain. Similar to the findings in this study, the Coronary Artery Risk Development in Young Adults study did not reveal significant associations between cFGF23 and myocardial strain parameters.^39^ In MESA, iFGF23 was independently associated with abnormal LV global circumferential strain, LV mid-wall circumferential strain, and left atrial total emptying fraction on cardiac magnetic resonance at 10-year follow-up.^40^ MESA had a different study design from ours, however, because it followed patients for over a decade, authors were able to find a longitudinal association between iFGF23 and subclinical markers of cardiac dysfunction.^40^ Although differences in study design and participant population may account for the variance in findings, it is also possible that FGF23 is not independently associated with changes in early cardiac mechanics, especially in a population with low prevalence of CKD.

This study has important limitations. Small sample size may have limited us from finding significant associations between cFGF23 and iFGF23 and 2D-STE parameters. 2D-STE imaging preceded FGF23 measurements by approximately 2 years. Although data suggest that FGF23 levels are stable over time, especially in individuals with preserved kidney function, this remains a limitation to our study design.^41–43^ In addition, the delay from sample collection to specimen thawing for FGF23 measurement is also a limitation. Although 2D-STE is a novel technique for cardiac imaging that is useful in measuring early changes in cardiac systolic and diastolic function, it has inherent limitations. Accuracy of 2D-STE depends on image quality and frame rates. It is also limited by through-plane motion or the loss of some speckles that move out of the image plane.^44^ In addition, CHS used archived research videotapes to measure cardiac mechanics indices, and the values are not comparable with those obtained clinically.

This investigation expands the literature on the associations of FGF23 with CVD by focusing on early abnormalities in cardiac structure and function that are precursors to clinical HF events. Given the emphasis on risk-based identification of HF and the growing importance of prevention of HF in high-risk populations, identifying possible modifiable risk factors that are related to early changes in cardiac mechanics remains critically relevant. While we did not find significant associations between FGF23 and indices of cardiac mechanics, further studies in populations with more advanced CKD are indicated given the expanding literature demonstrating direct links between FGF23 and HF outcomes. Additional research is needed to better understand the progression from subclinical to clinical HF in patients with and without CKD and to elucidate novel, modifiable risk factors that can be targeted in future clinical trials.

## Data Availability

All data are included in the manuscript and/or supporting information.

## Supplemental Material

Supplemental Table 1. Demographics and clinical characteristics by total CHS cohort versus subcohort.

Supplemental Table 2. Demographics and clinical characteristics by quartiles of iFGF23 in the cardiac subcohort.
